# Hybrid *de novo* genome assembly of the Chinese herbal plant danshen (*Salvia miltiorrhiza* Bunge)

**DOI:** 10.1186/s13742-015-0104-3

**Published:** 2015-12-14

**Authors:** Guanghui Zhang, Yang Tian, Jing Zhang, Liping Shu, Shengchao Yang, Wen Wang, Jun Sheng, Yang Dong, Wei Chen

**Affiliations:** Yunnan Research Center on Good Agricultural Practice for Dominant Chinese Medicinal Materials, Yunnan Agricultural University, Kunming, 650201 China; Key Laboratory of Pu-erh Tea Science, Ministry of Education, Yunnan Agricultural University, Kunming, 650201 China; College of Life Sciences, Jilin University, Changchun, 130012 China; College of Life Sciences, Huazhong University of Science and Technology, Wuhan, 430074 China; College of Life Science, Kunming University of Science and Technology, Kunming, 650504 China; State Key Laboratory of Genetic Resources and Evolution, Kunming Institute of Zoology, Chinese Academy of Sciences, Kunming, 650223 China; Yunnan Research Institute for Local Plateau Agriculture and Industry, Kunming, 650201 China

**Keywords:** *Salvia miltiorrhiza* Bunge, Illumina sequencing, PacBio sequencing, High heterozygous genome assembly

## Abstract

**Background:**

Danshen (*Salvia miltiorrhiza* Bunge), also known as Chinese red sage, is a member of Lamiaceae family. It is valued in traditional Chinese medicine, primarily for the treatment of cardiovascular and cerebrovascular diseases. Because of its pharmacological potential, ongoing research aims to identify novel bioactive compounds in danshen, and their biosynthetic pathways. To date, only expressed sequence tag (EST) and RNA-seq data for this herbal plant are available to the public. We therefore propose that the construction of a reference genome for danshen will help elucidate the biosynthetic pathways of important secondary metabolites, thereby advancing the investigation of novel drugs from this plant.

**Findings:**

We assembled the highly heterozygous danshen genome with the help of 395 × raw read coverage using Illumina technologies and about 10 × raw read coverage by using single molecular sequencing technology. The final draft genome is approximately 641 Mb, with a contig N50 size of 82.8 kb and a scaffold N50 size of 1.2 Mb. Further analyses predicted 34,598 protein-coding genes and 1,644 unique gene families in the danshen genome.

**Conclusions:**

The draft danshen genome will provide a valuable resource for the investigation of novel bioactive compounds in this Chinese herb.

**Electronic supplementary material:**

The online version of this article (doi:10.1186/s13742-015-0104-3) contains supplementary material, which is available to authorized users.

## Data description

### Danshen genomic DNA sequencing on Illumina platforms

Genomic DNA was extracted from the leaf tissues of a single danshen plant using the cetyltrimethylammonium bromide (CTAB) method. Paired-end libraries with insert sizes ranging from 350 to 900 bp were constructed using NEBNext Ultra II DNA Library Prep Kit for Illumina (NEB, USA), and mate pair libraries with insert sizes of 5 and 10 kb were constructed using Illumina Nextera Mate Pair Library Preparation Kit (Illumina, USA). Of all the constructed libraries, two with insertion sizes of 400 and 550 bp were sequenced on a MiSeq platform (Illumina, USA) using the PE-300 module [1], while the rest were sequenced on a HiSeq 2500 platform (Illumina, USA) using either a PE-100 or PE-90 module. Sequencing statistics for all libraries are outlined in Additional file 1: Table S1. In total, about 1.88 billion reads were generated on Illumina platforms, representing ~225 Gb of raw data. For the data filtering process, we discarded reads that met either of two following criteria: (1) which contained five or more low quality bases (quality shift value = 33); (2) which contained two percent or more ambiguous bases. With the removal of low quality and duplicated reads, ~147 Gb of clean data were obtained for the *de novo* assembly of the danshen genome.

### Single-molecule super-long reads sequencing on PacBio platform

Because the danshen genome is highly heterozygous, we sequenced super-long reads on a PacBio RS II platform (Pacific Biosciences, USA) to facilitate the subsequent *de novo* genome assembly process [2]. In brief, the Qiagen DNeasy Plant Mini Kit (Qiagen, Germany) was used to extract genomic DNA from the leaf tissues of a danshen plant. A total of 8 μg sheared DNA was used to construct a PacBio RS reads library with an insert size of 10 kb. The libraries were sequenced in 16 single-molecule real-time DNA sequencing cells using P5 polymerase, C3 chemistry combination, and a data collection time of 240 min per cell. The process yielded ~6.5 Gb of initially filtered PacBio data (read quality R 0.7, read length R 700 bp), which consisted of 2,359,223 reads with an average read length of 2,756 bp (Additional file 1: Figure S1).

### Estimation of danshen genome size and sequencing coverage

All ~147 Gb of clean reads obtained from the Illumina platforms were subjected to the 23-mer frequency distribution analysis with Jellyfish [3]. Analysis parameters were set at −t 50 −k 23, and the final result was plotted as a frequency graph (Additional file 1: Figure S2). Two distinctive modes could be observed from the distribution curve: (1) the higher peak at a depth of 54 demonstrated the high heterozygosity of the danshen genome; and (2) the lower peak provided a peak depth of 109 for the estimation of its genome size. Since the total number of *k*-mers was 70,389,985,464, the danshen genome size was calculated to be approximately 645.78 Mb, using the formula: genome size = *k*-mer_Number/Peak_Depth. This indicated that the sequenced Illumina reads (225 Gb) and PacBio RS reads (6.5 Gb) gave ~395 × and ~10 × coverage, respectively.

### Hybrid *de novo* genome assembly

A hybrid genome assembly pipeline was employed to cope with the high heterozygosity observed in the danshen genome (Additional file 1: Figure S3). First, we generated the Illumina-based *de novo* genome assembly using Platanus [4], which resulted in a draft danshen genome of 641 Mb, with a contig N50 size of 297 bp and a scaffold N50 size of 67.5 kb. Then, all PacBio RS reads were used to fill the gap by SSPACE-long reads [5], yielding a contig N50 size of 82.8 kb. Finally, the size of scaffold N50 was extended to 1.2 Mb after using SOAPdenovo scaffolding and Gapcloser [6]. Using this assembly pipeline, we obtained a final draft danshen genome of 641 Mb, with a contig N50 size of 82.8 kb and scaffold N50 size of 1.2 Mb.

### Evaluation of the completeness of danshen genome assembly

We compared the danshen genome assembly against a set of 248 ultra-conserved core eukaryotic genes using CEGMA [7] to evaluate the quality of the final assembly. The result showed that 221 of 248 genes could be fully annotated (89.11 % completeness, see Additional file 1: Table S2), and 238 of 248 genes met the criterion for partial annotation (95.97 % completeness).

### Repeat annotation of the danshen genome assembly

Analysis of the danshen genome using Tandem Repeat Finder [8] identified ~33.1 Mb tandem repeats, accounting for 5.02 % of the genome assembly (Additional file 1: Table S3). For the transposable element annotation, RepeatMasker [9] and RepeatProteinMasker [9] were used against Repbase [10] to identify known repeats in the danshen genome. In addition, RepeatModeler [9] and LTR FINDER [11] were used to identify *de novo* evolved repeats in the assembled genome. The combined results show that the total number of repeat sequences made up 53.58 % of the danshen genome assembly.

### Gene annotation

We conducted gene annotations for the danshen genome using a variety of methods, including EST and transcriptome-based predictions, *de novo* predictions, and homology-based predictions.

RNA-seq data sets for danshen leaf, root and flower tissues were obtained from the National Center for Biotechnology Information (NCBI) database (SRX388784, SRX371961, SRX370399 and 10,494 ESTs), and subsequently used for *de novo* assembly of the transcriptome. We aligned all RNA reads to the danshen genome using TopHat [12], assembled the transcripts with Cufflinks [12] using default parameters, and predicted the open reading frames to obtain reliable transcripts with hidden Markov model (HMM)-based training parameters. The transcriptome assembly resulted in 46–68 Mb of data for different tissues, totaling over 110,000 transcripts. After the removal of redundant data, we obtained 40,700 transcripts with an average length of 2,606 bp. Additionally, a 10,494 danshen EST data set was blasted using the assembled genome and identified 3,974 transcripts with an average length of 1,596 bp.

For *de novo* predictions, we performed Augustus [13] and GenScan [14] analysis on the repeat-masked danshen genome, with parameters trained from *Arabidopsis thaliana*. The resultant data sets were filtered with the removal of partial sequences and genes of <150 bp coding DNA sequences (CDS) length. These two methods yielded 27,753 and 32,305 transcripts, respectively.

For homology-based predictions, protein sequences of *Eucalyptus grandis*, *Sesamum indicum*, and *Ricinus communis* from Phytozome v9.1 database, and protein sequences of all 39 plants in the Ensembl Plants database (release 29) were individually mapped onto the danshen genome using TBLASTN with the same cutoff E-value at 1e-5. Homologous genome sequences were aligned against the matching proteins using GeneWise [15] for accurate spliced alignments. The number of transcripts from homology-based predictions ranged between 13,423 (*Oryza sativa*) and 29,158 (*Solanum tuberosum*). The average length of the transcript ranged from 1,603 to 2,891 bp.

We analyzed the gene annotation results from all *de novo* and homology-based predictions using EVidenceModeler and PASA [16] to produce a consensus protein-coding gene set. This gene set was finalized by filtering out those genes containing one exon, which were supported only by the transcriptome and EST-derived data. In sum, the danshen genome contains 34,598 protein-coding genes with an average CDS length of 1,078 bp (Additional file 1: Table S4).

### Ortholog clustering and gene family clustering analyses

Ortholog clustering analysis and gene family clustering analysis were performed using OrthoMCL [17] on all the protein-coding genes of danshen and *Arabidopsis thaliana*, *Eucalyptus grandis*, *Sesamum indicum*, *Solanum lycopersicum*, *Vitis vinifera*, *Oryza sativa*, *Populus trichocarpa*, *Solanum tuberosum*, and *Ricinus communis*. In brief, the 34,598 protein-coding genes in danshen are comprised of 3,454 single-copy orthologs, 10,121 multiple-copy orthologs, 5,725 unique paralogs, 8,689 other orthologs, and 6,609 unclustered genes (Additional file 1: Figure S4). Additionally, a total of 13,176 gene families were identified in the danshen genome. Among these gene families, 1,644 were unique gene families (Additional file 1: Table S5).

### Expression of genes related to flavonoid biosynthesis in different tissues

The biosynthetic pathway of rosmarinic acid from L-phenylalanine is of great importance for the production of many active ingredients in danshen [18]. Using the genes involved in the flavonoid biosynthetic pathway in *A. thaliana* as reference [19], candidate homologous genes in danshen were identified by matching their protein sequences to those of *A. thaliana* genes using BLASTP. FastTree [20] was then used to construct phylogenetic trees of candidate genes to identify the best match. We subsequently checked the expression levels of the identified danshen genes in different tissues. For example, *PHENYLALANINE AMMONIA*-*LYASE*, *CINNAMIC ACID 4*-*HYDROXYLASE*, and *HYDROXYCINNAMATE*: *COENZYME A LIGASE*, which are central to the phenylpropanoid pathway, showed higher expression levels in the danshen root tissues.

## Availability of supporting data

The assembly and annotation of the danshen genome are available at http://www.herbal-genome.cn. The sequencing reads of each sequencing library have been deposited at NCBI with the Project ID SRP059710. Supporting data including annotations and scaffolds is also available in the *GigaScience* database, GigaDB [21]. All supplementary figures and tables are provided in Additional file 1.

## Additional file

Additional file 1: Supplemental tables and figures.
**Table S1.** Raw sequencing statistics from the Illumina platform. **Table S2.** Evaluation of the completeness of the danshen genome based on 248 core eukaryotic genes. **Table S3.** Transposable element annotation statistics for the danshen genome. **Table S4.** Gene annotation statistics for the danshen genome. **Table S5.** Statistics for gene family clustering analysis. **Figure S1.** Frequency counts of all PacBio reads per read length. **Figure S2.** Frequency distribution of the 23-mer graph. **Figure S3.** Assembly pipeline for the danshen genome combining Illumina data and PacBio data. **Figure S4.** Ortholog clustering analysis of the protein-coding genes among *Arabidopsis thaliana*, *Salvia miltiorrhiza*, *Eucalyptus grandis*, *Oryza sativa*, *Populus trichocarpa*, *Ricinus communis*, *Sesamum indicum*, *Solanum lycopersicum*, *Solanum tuberosum*, *Vitis vinifera*. (DOCX 428 kb)

## Abbreviations

CDS: Coding DNA sequence
CTAB: Cetyltrimethylammonium bromide
EST: Expressed sequence tag
NCBI: National Center for Biotechnology Information
