# Supplementary material for: Hybrid de novo genome assembly of the Chinese herbal plant danshen (Salvia miltiorrhiza Bunge)
Source: Gigascience. 2015 Dec 14;4:62. doi: 10.1186/s13742-015-0104-3 (PMC4678694; doi:10.1186/s13742-015-0104-3)
Supplement: Additional file 1: Supplemental tables and figures. — Table S1. Raw sequencing statistics from the Illumina platform. Table S2. Evaluation of the completeness of the danshen genome based on 248 core eukaryotic genes. Table S3. Transposable element annotation statistics for the danshen genome. Table S4. Gene annotation statistics for the danshen genome. Table S5. Statistics for gene family clustering analysis. Figure S1. Frequency counts of all PacBio reads per read length. Figure S2. Frequency distribution of the 23-mer graph. Figure S3. Assembly pipeline for the danshen genome combining Illumina data and PacBio data. Figure S4. Ortholog clustering analysis of the protein-coding genes among Arabidopsis thaliana, Salvia miltiorrhiza, Eucalyptus grandis, Oryza sativa, Populus trichocarpa, Ricinus communis, Sesamum indicum, Solanum lycopersicum, Solanum tuberosum, Vitis vinifera. (DOCX 428 kb) [file 13742_2015_104_MOESM1_ESM.docx]

**Additional tables**

**Table S1. Raw sequencing statistics from the Illumina platform.**

|  | **Insert size (bp)** | **Reads length (bp)** | **No. of reads** | **Raw reads (Gb)** | **Clean reads (Gb)** |
| --- | --- | --- | --- | --- | --- |
| Miseq | 400 | PE300 | 36,779,442 | 11.00 | 10.24 |
| Miseq | 550 | PE300 | 52,336,798 | 15.70 | 14.60 |
| Hiseq2500 | 350 | PE100 | 411,335,450 | 41.13 | 32.97 |
| Hiseq2500 | 350 | PE100 | 453,018,138 | 45.30 | 34.29 |
| Hiseq2500 | 550 | PE100 | 456,429,074 | 45.64 | 31.76 |
| Hiseq2500 | 900 | PE100 | 367,837,280 | 36.78 | 14.27 |
| Hiseq2500 | 5,000 | PE90 | 337,573,672 | 30.38 | 4.01 |
| Hiseq2500 | 10,000 | PE90 | 324,020,242 | 29.16 | 5.06 |
| **Total Summary** | | | **1,878,528,688** | **255.09*** | **147.20** |

***** Total raw reads represents approximately 395 × coverage of the danshen genome.

**Table S2. Evaluation of the completeness of the danshen genome based on 248 core eukaryotic genes.**

|  | **Number of CEGs** | **Completeness (%)** | **Number of CEGs and orthologs** | **Orthologs per CEG** | **% CEGS with ≥ 1 ortholog** |
| --- | --- | --- | --- | --- | --- |
| **Complete** | **221** | **89.11** | **443** | **2.00** | **55.66** |
| Group 1 | 57 | 86.36 | 97 | 1.70 | 43.86 |
| Group 2 | 49 | 87.50 | 86 | 1.76 | 40.82 |
| Group 3 | 53 | 86.89 | 111 | 2.09 | 60.38 |
| Group 4 | 62 | 95.38 | 149 | 2.40 | 74.19 |
| **Partial** | **238** | **95.97** | **531** | **2.23** | **62.61** |
| Group 1 | 62 | 93.94 | 118 | 1.90 | 48.39 |
| Group 2 | 52 | 92.86 | 102 | 1.96 | 50.00 |
| Group 3 | 60 | 98.36 | 143 | 2.38 | 71.67 |
| Group 4 | 64 | 98.46 | 168 | 2.62 | 78.12 |

**Table S3. Transposable element annotation statistics for the danshen genome**

| **Methods** | **Repeat size (bp)** | **Percent of genome (%)** |
| --- | --- | --- |
| Tandem Repeat Finder | 33,102,154 | 5.02 |
| RepeatMasker | 409,776 | 0.06 |
| RepeatProteinMasker | 83,864,539 | 12.71 |
| *De novo* | 335,698,178 | 50.88 |
| **Merged data** | **353,513,348** | **53.58** |

**Table S4. Gene annotation statistics for the danshen genome.**

| **Methods** | **Number of transcript** | **Average transcript length (bp)** | **Average CDS length (bp)** | **Average exon per gene** | **Average exon length (bp)** | **Average intron length (bp)** |
| --- | --- | --- | --- | --- | --- | --- |
| **RNA-seq** | 40,700 | 2,606 | 1,163 | 4 | 288 | 474 |
| **EST** | 3,974 | 1,596 | 467 | 2 | 188 | 759 |
| ***De novo*** |  |  |  |  |  |  |
| AUGUSTUS | 27,753 | 4,316 | 1,181 | 6 | 207 | 665 |
| GenScan | 32,305 | 2,791 | 551 | 3 | 157 | 896 |
| **Homolog *** |  |  |  |  |  |  |
| *Arabidopsis thaliana* | 15,915 | 2,520 | 1,247 | 5 | 227 | 338 |
| *Eucalyptus grandis* | 17,187 | 2,712 | 1,290 | 6 | 225 | 354 |
| *Sesamum indicum* | 28,395 | 2,115 | 1,123 | 4 | 252 | 348 |
| *Solanum lycopersicum* | 26,846 | 1,966 | 1,056 | 4 | 245 | 339 |
| *Vitis vinifera* | 17,565 | 2,604 | 1,213 | 6 | 213 | 345 |
| *Oryza sativa* | 13,423 | 2,891 | 1,384 | 6 | 250 | 392 |
| *Populus trichocarpa* | 20,423 | 2,332 | 1,185 | 5 | 232 | 337 |
| *Solanum tuberosum* | 29,158 | 1,603 | 976 | 4 | 275 | 326 |
| *Ricinus communis* | 19,109 | 2,266 | 1,132 | 5 | 224 | 334 |
| 33 other plants | 20,945 | 2,183 | 1,103 | 4 | 273 | 425 |
| **EVidenceModeler** | **34,598** | **4,166** | **1,078** | **5** | **200** | **597** |

***** All 39 species in the Ensembl Plants database (release 29) were used. *E. grandis, S. indicum,* and *R. communis* were obtained from Phytozome.

**Table S5. Statistics for gene family clustering analysis.**

| **Species** | **Total gene number** | **No. of genes in families** | **Unclustered genes** | **No. of gene families** | **No. of unique gene families** | **Average gene per family** |
| --- | --- | --- | --- | --- | --- | --- |
| *Arabidopsis thaliana* | 35,395 | 31,704 | 3,691 | 13,517 | 1,184 | 2.35 |
| *Salvia miltiorrhiza* | 34,598 | 27,989 | 6,609 | 13,176 | 1,644 | 2.12 |
| *Eucalyptus grandis* | 36,368 | 28,929 | 7,439 | 13,717 | 815 | 2.11 |
| *Oryza sativa* | 42,132 | 29,472 | 12,660 | 13,553 | 2,474 | 2.17 |
| *Populus trichocarpa* | 45,787 | 37,739 | 8,048 | 15,334 | 1,150 | 2.46 |
| *Ricinus communis* | 31,221 | 20,783 | 10,438 | 14,595 | 781 | 1.42 |
| *Sesamum indicum* | 27,161 | 23,663 | 3,498 | 13,027 | 401 | 1.82 |
| *Solanum lycopersicum* | 34,730 | 26,421 | 8,309 | 16,487 | 561 | 1.60 |
| *Solanum tuberosum* | 35,119 | 28,885 | 6,234 | 15,540 | 628 | 1.86 |
| *Vitis vinifera* | 29,936 | 22,535 | 7,401 | 13,992 | 716 | 1.61 |

**Additional Figures**

**Figure S1. Frequency counts of all PacBio reads per read length.**

**Figure S2. Frequency distribution of the 23-mer graph.**


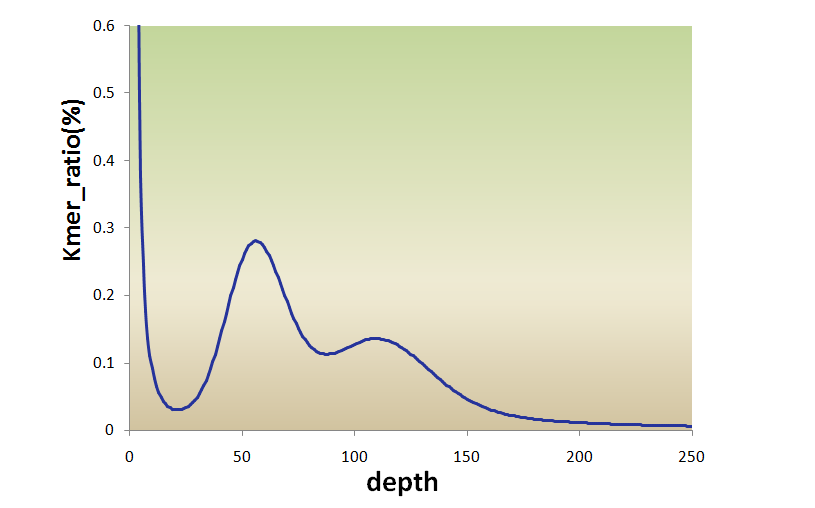


**(X)**

**Figure S3. Assembly pipeline for the danshen genome combining Illumina data and PacBio data.**


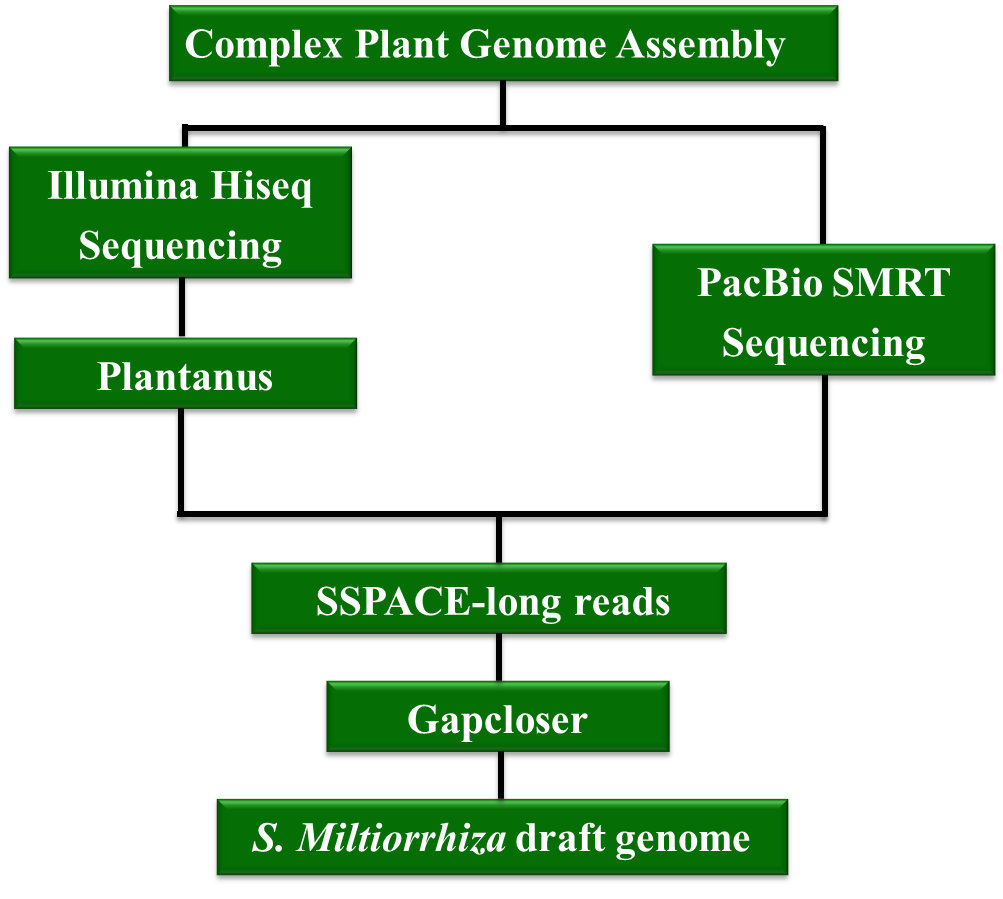


**Figure S4. Ortholog clustering analysis of the protein-coding genes among *Arabidopsis thaliana, Salvia miltiorrhiza, Eucalyptus grandis, Oryza sativa, Populus trichocarpa, Ricinus communis, Sesamum indicum, Solanum lycopersicum, Solanum tuberosum, Vitis vinifera.***
